# Partners of cancer patients consult their GPs significantly more often with both somatic and psychosocial problems

**DOI:** 10.3109/02813432.2013.861153

**Published:** 2013-12

**Authors:** Marianne Heins, François Schellevis, Mieke Rijken, Gé Donker, Lucas van der Hoek, Joke Korevaar

**Affiliations:** ^1^Netherlands Institute for Health Services Research (NIVEL), The Netherlands; ^2^Department of General Practice/EMGO Institute for Health and Care Research, VU University Medical Centre, Amsterdam

**Keywords:** Cancer partner, caregiver, general practice, the, Netherlands

## Abstract

**Objective:**

Partners of cancer patients experience psychological distress and impaired physical health around and after the diagnosis of cancer. It is unknown whether these problems are presented to the general practitioner (GP). This study aimed to establish partners’ GP use around the diagnosis of cancer.

**Design:**

Cohort study.

**Setting:**

Primary care.

**Subjects:**

Partners of 3071 patients with breast, prostate, colorectal, or lung cancer were included. Patients were diagnosed in 2001–2009 and were alive at least two years after diagnosis.

**Main outcome measures:**

Number of GP contacts and health problems in partners between six months before and two years after diagnosis.

**Results:**

In the first six months after diagnosis, partners’ GP use was similar to baseline (18 to six months before diagnosis). Between six and 24 months after diagnosis, GP use was increased in partners of patients with breast, prostate, and colorectal cancer, an increase of 31% (p = 0.001), 26% (p = 0.001), and 19% (p = 0.042), respectively. In partners of patients with breast cancer and colorectal cancer, GP use was increased for both somatic and psychosocial symptoms. In partners of prostate cancer patients, an increase was seen in somatic symptoms, whereas in partners of lung cancer patients, GP use was only increased for psychosocial symptoms. “Problems with the illness of the partner” was a frequently recorded reason for contact in the first six months after diagnosis.

**Conclusion:**

GP use of partners of cancer patients is increased 6–24 months after diagnosis, but health problems vary between cancer types. GPs should be alert for somatic and psychosocial problems in partners of cancer patients.

- Partners of cancer patients experience increased psychological distress and have more hospital admissions during the first two years after diagnosis than before the cancer diagnosis.
- Partners of patients with cancer have more GP contacts in the first two years after the diagnosis.
- Partners of cancer patients consult their GP not only with increased psychological problems, but also more often with somatic symptoms.

## Introduction

Cancer not only affects patients, but also family, friends, and caregivers. Studies found psychological problems in 20–30% of cancer patients’ partners [1–6] increasing to 30–50% in the palliative phase [7–9]. Psychological distress in partners may even be higher than in patients themselves [10,11]. Females report more distress than males, regardless of their patient/partner status [12]. Other factors related to distress include previous psychiatric illness, negative perception of the disease, lack of social support, and partner relationship problems [13].

Physical health may also be affected. Informal caregivers of cancer patients, mostly partners [14], experience more fatigue, pain, sleep problems, and eating disorders [15]. Physical health is worse when they perceive the illness as more serious and burden of care giving as high. Objective measures, such as recurrence status or time spent providing care, are not related to physical health [16], suggesting that psychological burden rather than physical burden of care giving is worsening physical health.

Psychological and somatic problems may lead to increased health care use. Indeed, partners of patients with colon and lung cancer are more often hospitalized compared with persons of the same age and sex [17], especially for psychiatric problems. In countries with a strong primary care system, the general practitioner (GP) is most likely the first to be consulted for health problems. Cancer patients’ partners indeed have more GP visits in the period around the patient's death [18,19], but the period around the diagnosis has not been studied yet.

In the months before diagnosis, GP use might be decreased because concerns regarding the patient's health prevail. GP use might only increase months after diagnosis, when the first turmoil has passed. Furthermore, it is unknown for which health problems partners consult their GP. Knowing this may help GPs to identify and discuss partners’ health problems at an early stage. We therefore investigated GP use of partners of adult cancer patients around the diagnosis.

## Material and methods

### Study population

Data were derived from the Netherlands Information Network of General Practice (LINH), a network of about 90 practices representative of the Netherlands, holding data on contacts, diagnoses, and prescriptions of approximately 350 000 individuals [20]. Diagnostic coding is accurate [21]. In the Netherlands, all inhabitants are obligatorily insured for standard medical care, including GP visits, and are listed with a GP, who is gatekeeper to secondary care [22].

We first selected index patients, i.e. adult patients diagnosed with breast, prostate, colorectal, or lung cancer (ICPC codes X76, Y77, D75, and R84) between 2001 and 2009. Cancer types were chosen based on their high incidence. We excluded patients who died within two years, as we focused on the effect of the diagnosis, not the palliative phase and patient's death. We excluded practices providing data for less than 48 weeks per year or lacking over 50% of ICPC codes (including non-informative ICPC codes A97 [no disease] and A99 [other unspecified disease]). The percentage of missing ICPC codes in LINH decreased from 30% in 2002 to 13% in 2009.

Partners were identified indirectly. Within the Electronic Medical Record (EMR), every household, i.e. address, receives a unique number. We selected those living in the index patients’ households at diagnosis. Households where someone was diagnosed with cancer before or within two years of the index patient and households exceeding six persons (possibly nursing homes or other institutions) were excluded. We considered someone a partner when: the age difference from the patient was < 20 years, to exclude children or parents, and the age was > 25, to exclude siblings of young patients. Households were excluded when multiple partners were identified.

Partners’ GP use (office visits, home visits, telephone consultations with a GP or practice nurse) and related ICPC codes were extracted for the period between 18 months before to two years after diagnosis. Months in which the practice did not provide data or the partner was not registered were excluded.

The study was conducted according to the precepts of the Helsinki Declaration, Dutch privacy legislation and regulations of the Dutch Data Protection Authority. According to Dutch legislation, obtaining informed consent is not necessary for observational research with anonymized patient data.

### Clusters

To test whether GP use was altered for specific types of health problems, ICPC codes were grouped into five clusters: “acute symptoms” (e.g. pain symptoms, injuries), “infections” (e.g. upper respiratory, urinary tract), “chronic diseases” (e.g. diabetes, hypertension), “psychosocial problems” (chapter P and Z), and “other” (all other codes). These clusters have been used in studies of cancer survivors [23,24].

### Statistical analyses

We first established partners’ baseline GP use, i.e. mean monthly contact rate between 18 and six months before diagnosis. Next, for each month between six months before and two years after diagnosis, we divided the monthly contact rate by baseline GP use. To improve presentation of graphs, we used three-week non-weighted moving averages (i.e. average of the contact rate in the month itself, the preceding and the succeeding month, see for an example [25]).

We compared the contact rate with baseline GP use using negative binomial regression. Moving averages were not used for these analyses. To limit the number of tests, we composed three time-intervals: the six months before diagnosis, the six months after diagnosis, and months 6–24 after diagnosis. Regression models included monthly contact rate as dependent and three dummy time-interval variables as independent variables. Regression parameters were expressed as incidence rate ratios (IRRs), i.e. the ratio of the contact rate and baseline GP use.

We repeated this procedure for each cluster of health problems. GPs could register multiple diagnoses per contact, so one contact could be related to multiple clusters. Additionally, for the periods of 0–6 and 6–24 months after diagnosis, we established which ICPC codes were recorded more frequently compared with baseline.

Analyses were stratified by cancer type and performed with STATA^®^ SE 11.2. We corrected for multiple testing using the FDR method [26]. This method accounts for the fact that p-values just below 0.05 are more likely to be false positives than smaller p-values.

## Results

### Patients and partners

Between 2001 and 2009, 2414 patients were diagnosed with breast cancer, 1197 with prostate cancer, 1387 with colorectal cancer, and 1167 with lung cancer. Of these patients, 91 were living in households exceeding six persons, 2054 patients had no partner, and for 30 patients multiple (plausible) partners were identified. For 396 patients another household member had been diagnosed with cancer or was diagnosed within two years. Of the remaining 3594 patients, 523 died within two years (57 breast, 80 prostate, 120 colorectal, and 266 lung cancer patients). Baseline characteristics are presented in Table I.

**Table I.** Baseline characteristics of index patients and partners.

		Breast (n = 1288)	Prostate (n = 705)	Colorectal (n = 649)	Lung (n = 429)
Index patients	Age	56.1 (11.6)	71.0 (8.7)	65.7 (11.4)	65.3 (10.8)
	Sex (male (%))	0 (0%)	705 (100%)	394 (61%)	308 (72%)
Partners	Age	57.8 (12.0)	67.7 (8.9)	64.3 (11.7)	63.5 (10.9)
	Sex (male (%))	1269 (99%)	3 (0%)	255 (39%)	123 (29%)
	GP contacts per year^1^	2.5 (3.6)	4.5 (5.4)	4.0 (5.1)	3.9 (4.9)
	*Acute symptoms*	0.7 (1.4)	1.1 (1.9)	1.1 (1.9)	1.0 (1.5)
	*Infections*	0.3 (0.8)	0.4 (1.1)	0.4 (1.2)	0.4 (1.2)
	*Chronic disease*	0.5 (1.6)	1.0 (2.0)	0.9 (2.0)	0.9 (2.1)
	*Psychosocial*	0.1 (0.5)	0.2 (1.0)	0.2 (0.9)	0.1 (0.6)

Notes: Numbers are mean (standard deviation) or absolute number (percentage). ^1^In the period between 18 and six months before diagnosis.

### Overall GP use

**Figure 1.**
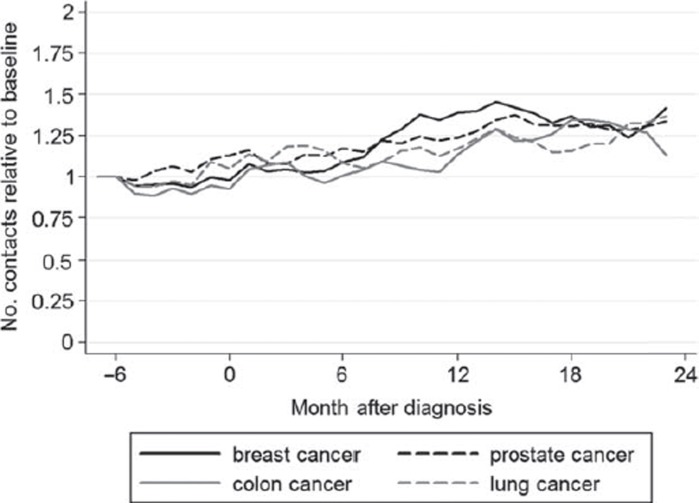
Relative number of GP contacts in cancer patients’ partners from six months before to 24 months after diagnosis by cancer type compared with baseline (18 to six months before diagnosis – set at 1.0).

As can be seen in Figure 1, partners’ GP use in the six months before and after diagnosis was similar to baseline, which was confirmed by regression analysis. Between six and 24 months after diagnosis, GP use was higher in partners of patients with breast, prostate, and colorectal cancer (IRR = 1.31 (95% CI 1.18–1.45, p = 0.001), 1.26 (95% CI 1.13–1.42, p = 0.001), and 1.18 (95% CI 1.04–1.33, p = 0.042)). In partners of lung cancer patients overall GP use did not increase (IRR = 1.19 (95% CI 1.02–1.38, p = 0.08)).

### PC use by cluster

**Figure 2.**
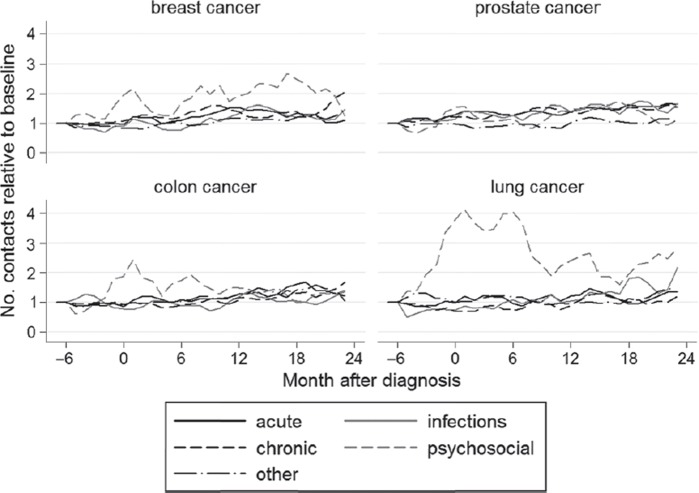
Types of diagnoses (ICPC codes) during GP contacts in cancer patients’ partners six months before to 24 months after diagnosis compared with baseline (18 to six months before diagnosis – set at 1.0).

As can be seen in Figure 2, in partners of breast cancer patients GP use for psychosocial and acute symptoms and chronic diseases increased between six and 24 months after diagnosis (IRR = 2.03 (95% CI 1.30–3.17, p = 0.01), 1.30 (95% CI 1.1–1.50, p = 0.001), and 1.44 (95% CI 1.16–1.78, p = 0.008)). In partners of prostate cancer patients GP use for acute symptoms increased 0–6 months after diagnosis (IRR = 1.40 (95% CI 1.18–1.66, p = 0.001)), and GP use for acute symptoms, chronic diseases, and infections increased between six and 24 months after diagnosis (IRR = 1.45 (95% CI 1.22–1.73, p = 0.001), 1.45 (95% CI 1.21–1.75, p = 0.001), and 1.46 (95% CI 1.14–1.86, p = 0.01)). In partners of colorectal cancer patients GP use for psychosocial problems increased 0–6 months after diagnosis (IRR = 1.83 (95% CI 1.20–2.78, p = 0.03)), and GP use for acute symptoms between six and 24 months after diagnosis (IRR = 1.26 (95% CI 1.05–1.51, p = 0.048)). In partners of lung cancer patients GP use for psychosocial symptoms increased in both periods (IRR = 3.85 (95% CI 2.15–6.87, p = 0.001) for 0–6 months and IRR = 2.50 (95% CI 1.33–4.71, p = 0.03) between six and 24 months after diagnosis).

### Specific problems

When focusing on specific health problems that were more frequent in the first six months after diagnosis, “problems with illness of partner” (ICPC Z14) was repeatedly the first or second most frequently recorded diagnosis (Table IIa). Between six and 24 months after diagnosis, common health problems like hypertension and cough were most prevalent, although “problems with illness of partner” was still the second most common diagnosis in partners of lung cancer patients (Table IIb) and the fifth most common in partners of prostate cancer patients.

**Table IIa.** Frequency of specific diagnoses (ICPC codes) that were more prevalent in partners 0–6 months after diagnosis in index patients.

	ICPC		No. of patients	No. of contacts per 1000 patient years	IRR (95% CI)
Breast cancer	K86	Hypertension uncomplicated	82	290.1	1.6 (1.4–1.9)
	T90	Diabetes	47	173.7	1.3 (1.1–1.6)
	H81	Excessive ear wax	25	52.4	1.5 (1.1–2.2)
Prostate cancer	L03	Low back pain	19	68.2	1.9 (1.0–3.8)
	Z14	Problem with illness of partner	12	53.5	19.2 (5.2–160.4)
Colorectal cancer	Z14	Problem with illness of partner	22	117.7	5.4 (2.5–13.6)
	Z06	Sleep problem	18	68.7	2.5 (1.2–6.1)
Lung cancer	Z14	Problem with illness of partner	21	142.4	24.3 (4.3–966.8)

Note: ^1^ICPC codes with significantly more contacts compared with baseline and a minimum of 10 patients, ordered by number of contacts per 1000 patient years.

**Table IIb.** Frequency of specific diagnoses (ICPC codes) that were more prevalent in partners 6–24 months after diagnosis in index patients.

	ICPC		No. of patients	No. of contacts per 1000 patient years	IRR (95% CI)
Breast cancer	K86	Hypertension uncomplicated	127	308.6	1.9 (1.7–2.3)
	H81	Excessive ear wax	78	74.3	1.6 (1.2–2.3)
	T90	Diabetes	66	211.6	1.7 (1.4–2.0)
Prostate cancer	R74	Upper respiratory infection acute	39	68.5	2.6 (1.3–5.8)
	D06	Localized abdominal pain	37	90.1	2.4 (1.2–5.3)
	L03	Low back pain	30	60.9	1.9 (1.1–3.7)
Colorectal cancer	T90	Diabetes	43	354.1	1.3 (1.1–1.6)
	R78	Acute bronchitis	32	79.0	1.9 (1.2–3.4)
	P06	Sleep problem	21	53.3	2.1 (1.0–4.9)
Lung cancer	L99	Other musculoskeletal disease	20	88.4	2.5 (1.1–6.8)
	Z14	Problem with illness of partner	12	40.6	9.3 (1.6–368.8)

Note: ^1^ICPC codes with significantly more contacts compared with baseline and a minimum of 10 patients, ordered by number of contacts per 1000 patient years.

## Discussion

This study shows that partners of patients with breast, prostate, and colorectal cancer have increased GP use between six and 24 months after diagnosis, compared with a baseline measurement. GP use only rises several months after diagnosis, presumably because concerns about the patient's health prevail in the period around diagnosis. Overall GP use does not seem to increase in partners of lung cancer patients. Particularly in this severe disease, less urgent health problems of the partner may become secondary to those of the patient.

Also the type of health problems recorded during consultations differs between cancer types. Partners of patients with breast and colorectal cancer have increased GP use for somatic and psychosocial symptoms. Partners of prostate cancer patients have increased GP use for somatic symptoms, whereas those of lung cancer patients show an almost fourfold increase for psychosocial symptoms. Differences may be related to the prognosis of the disease [27,28].

We used data from a nationally representative GP network, resulting in a large sample size without selection bias, but partners had to be identified through their household number and age difference with the patient. We may have included older siblings living together; however, their number will be low. We missed partners listed with different GPs or living at different addresses. It is unlikely that the impact of the cancer diagnosis is very different in these siblings/partners.

Our study did not include a control group, but we compared partners’ GP use around diagnosis with a baseline period. As the mean age of our study population was 57–68 years, a small increase in GP contacts could be expected over time. However, a previous study showed that the GP use in a non-cancer control group of similar age did not increase during a three-year period [29].

Data accuracy is likely to be high, as EMRs are used for reimbursement, and accuracy of ICPC coding is good [21]. GPs may have made coding errors, but these will be unlikely to differ systematically before and after someone's partner was diagnosed with cancer, so their impact seems limited. Recording of the cancer diagnosis in the EMR may be delayed but it is unlikely that the actual date of diagnosis lies in the baseline period.

Findings are likely to be generalizable to other countries with a strong primary care system, such as the UK, Denmark, and Canada. In countries with a less prominent primary care system, cancer patients’ partners may not have increased primary care use, as they may present their health problems to a specialist.

Our findings correspond with those of Sjovall et al., who found more secondary care psychiatric diagnoses in partners of colon and lung cancer patients [17]. Unlike the findings in our study, psychiatric diagnoses were also more common in partners of prostate cancer patients. Sjovall et al. included partners of patients who died during the study period, which may explain these differences [18]. Nakaya et al. found increased hospitalization for affective disorders in spouses of breast cancer patients, which was indeed higher when their wife died [30].

GP use increased between six and 24 months after the diagnosis, but visits related to the partner's illness are already seen in the first six months after diagnosis. Attention to partners’ health problems may therefore be needed shortly after the diagnosis. Partners may report problems more easily when possible consequences of the diagnosis for themselves have already been discussed. GPs should be alert for both somatic and psychosocial problems, although psychosocial problems may occur more often when the prognosis is poor.

In conclusion, partners’ GP use increases after the diagnosis of cancer. Based on our data, we cannot say how effectively GPs are responding to the partners’ needs. Future research is needed to find out whether they have unmet needs.

## References

[1] Compas BE, Worsham NL, Epping-Jordan JE, Grant KE, Mireault G, Howell DC (1994). When mom or dad has cancer: Markers of psychological distress in cancer patients, spouses, and children. Health Psychol.

[2] Hagedoorn M, Buunk BP, Kuijer RG, Wobbes T, Sanderman R (2000). Couples dealing with cancer: Role and gender differences regarding psychological distress and quality of life. Psychooncology.

[3] Rodrigue JR, Hoffmann RG (1994). Caregivers of adults with cancer: Multidimensional correlates of psychological distress. J Clin Psychol Med Settings.

[4] Ey S, Compas BE, Epping-Jordan JE, Worsham N (1999). Stress responses and psychological adjustment in patients with cancer and their spouses. J Psychosoc Oncol.

[5] Glasdam S, Jensen AB, Madsen EL, Rose C (1996). Anxiety and depression in cancer patients’ spouses. Psychooncology.

[6] Nijboer C, Triemstra M, Tempelaar R, Sanderman R, van den Bos GAM (1999). Determinants of caregiving experiences and mental health of partners of cancer patients. Cancer.

[7] Siegel K, Karus DG, Raveis VH, Christ GH, Mesagno FP (1996). Depressive distress among the spouses of terminally ill cancer patients. Cancer Pract.

[8] Kissane DW, Bloch S, Burns WI, McKenzie D, Posterino M (1994). Psychological morbidity in the families of patients with cancer. Psychooncology.

[9] Williamson GM, Schulz R (1995). Caring for a family member with cancer: Past communal behavior and affective reactions. J Appl Soc Psychol.

[10] Braun M, Mikulincer M, Rydall A, Walsh A, Rodin G (2007). Hidden morbidity in cancer: Spouse caregivers. J Clin Oncol.

[11] Eton DT, Lepore SJ, Helgeson VS (2005). Psychological distress in spouses of men treated for early-stage prostate carcinoma. Cancer.

[12] Hagedoorn M, Sanderman R, Bolks HN, Tuinstra J, Coyne JC (2008). Distress in couples coping with cancer: A meta-analysis and critical review of role and gender effects. Psychol Bull.

[13] Pitceathly C, Maguire P (2003). The psychological impact of cancer on patients’ partners and other key relatives: A review. Eur J Cancer.

[14] Nijboer C, Tempelaar R, Sanderman R, Triemstra M, Spruijt RJ, Van den Bos GA (1998). Cancer and caregiving: The impact on the caregiver's health. Psychooncology.

[15] Stenberg U, Ruland CM, Miaskowski C (2010). Review of the literature on the effects of caring for a patient with cancer. Psychooncology.

[16] Gregorio SW, Carpenter KM, Dorfman CS, Yang HC, Simonelli LE, Carson WE (2012). Impact of breast cancer recurrence and cancer-specific stress on spouse health and immune function. Brain Behav Immun.

[17] Sjovall K, Attner B, Lithman T, Noreen D, Gunnars B, Thome B (2009). Influence on the health of the partner affected by tumor disease in the wife or husband based on a population-based register study of cancer in Sweden. J Clin Oncol.

[18] King M, Vasanthan M, Petersen I, Jones L, Marston L, Nazareth I (2013). Mortality and medical care after bereavement: A general practice cohort study. PLoS One.

[19] Guldin MB, Jensen AB, Zachariae R, Vedsted P (2013). Healthcare utilization of bereaved relatives of patients who died from cancer: A national population-based study. Psychooncology.

[20] Stirbu-Wagner I, Dorsman SA, Visscher S, Davids R, Gravestein JV, Abrahamse H Landelijk Informatienetwerk Huisartsenzorg. Feiten en cijfers over huisartsenzorg in Nederland [Netherlands Information Network of General Practice. Facts and figures about general practice care the Netherlands].

[21] Van der Linden MW, Westert GP, Den Bakker DH, Schellevis FG (2004). Tweede Nationale Studie naar ziekten en verrichtingen in de huisartspraktijk. Klachten en aandoeningen in devbevolking en in de huisartspraktijk. [Second national study into diseases and actions in general practice].

[22] Schafer W, Kroneman M, Boerma W, van den BM, Westert G, Deville W (2010). The Netherlands: Health system review. Health Syst Transit.

[23] Groenewegen PP, de Bakker DH, van der Velden J (1992). Nationale studie naar ziekten en verrichtingen in de huisartspraktijk. Basisrapport verrichtingen in de huisartspraktijk [Dutch national survey of general practice. Report on interventions in general practice].

[24] Heins MJ, Korevaar JC, Rijken PM, Schellevis FG (2013). For which health problems do cancer survivors visit their general practitioner?. Eur J Cancer.

[25] Enserink R, Meijer A, Dijkstra F, van BB, van der Steen JT, Haenen A (2011). Absence of influenza A(H1N1) during seasonal and pandemic seasons in a sentinel nursing home surveillance network in the Netherlands. J Am Geriatr Soc.

[26] Benjamini Y, Hochberg Y (1995). Controlling the false discovery rate: A practical and powerful approach to multiple testing. J Royal Stat Soc.

[27] Zabora J, BrintzenhofeSzoc K, Curbow B, Hooker C, Piantadosi S (2001). The prevalence of psychological distress by cancer site. Psychooncology.

[28] Carmack Taylor C, Badr H, Lee J, Fossella F, Pisters K, Gritz E (2008). Lung cancer patients and their spouses: Psychological and relationship functioning within 1-month of treatment initiation. Ann Behav Med.

[29] Heins M, Korevaar J, Rijken M, Hoek vander L,, Schellevis F (2012). Determinants of primary health care use in cancer survivors. J Clin Oncol.

[30] Nakaya N, Saito-Nakaya K, Bidstrup PE, Dalton SO, Frederiksen K, Steding-Jessen M (2010). Increased risk of severe depression in male partners of women with breast cancer. Cancer.

